# The Cognitive, Ecological, and Developmental Origins of Self-Disturbance in Borderline Personality Disorder

**DOI:** 10.3389/fpsyt.2021.707091

**Published:** 2021-09-30

**Authors:** Axel Baptista, David Cohen, Pierre Olivier Jacquet, Valérian Chambon

**Affiliations:** ^1^Institut Jean Nicod, Département d'études Cognitives, ENS, EHESS, CNRS, PSL University, Paris, France; ^2^Service de Psychiatrie de l'Enfant et de l'Adolescent, GH Pitié-Salpêtrière Charles Foix, APHP.6, Paris, France; ^3^Université de Paris, Paris, France; ^4^Faculté de Médecine, Sorbonne Université, Paris, France; ^5^Institut des Systèmes Intelligents et de Robotique, Sorbonne Université, ISIR CNRS UMR 7222, Paris, France; ^6^Laboratoire de Neurosciences Cognitives & Computationnelles, Département d'études Cognitives, École Normale Supérieure, INSERM, PSL University, Paris, France; ^7^Institut du Psychotraumatisme de l'Enfant et de l'Adolescent, Centre Hospitalier de Versailles et Conseil départemental des Yvelines et des Hauts de Seine, Versailles, France

**Keywords:** borderline personality disorder, self, agency, social cognition, early life adversity, life history theory

## Abstract

Self-disturbance is recognized as a key symptom of Borderline Personality Disorder (BPD). Although it is the source of significant distress and significant costs to society, it is still poorly specified. In addition, current research and models on the etiology of BPD do not provide sufficient evidence or predictions about who is at risk of developing BPD and self-disturbance, and why. The aim of this review is to lay the foundations of a new model inspired by recent developments at the intersection of social cognition, behavioral ecology, and developmental biology. We argue that the sense of agency is an important dimension to consider when characterizing self-disturbances in BPD. Second, we address the poorly characterized relation between self-disturbances and adverse life conditions encountered early in life. We highlight the potential relevance of Life-History Theory—a major framework in evolutionary developmental biology—to make sense of this association. We put forward the idea that the effect of early life adversity on BPD symptomatology depends on the way individuals trade their limited resources between competing biological functions during development.

## Introduction

Clinical observations and experimental data, as well as a number of theoretical developments and international classifications, support the proposition that disturbances in self- and interpersonal functioning are fundamental to the psychopathology of Borderline Personality Disorder (BPD) (1–3). In addition, features of BPD, such as affective instability, impulsivity, and acts of self-harm most often occur in response to real or imagined interpersonal events.

BPD has a dramatic impact on global health and incurs a significant cost to society (2, 4). It is the most common personality disorder in clinical setting (33–49% in young inpatients) (4, 5), and psychosocial functioning is significantly impaired in affected individuals (6). Approximately 75% of BPD patients have a history of suicide attempt (7), and the risk of death by suicide in these patients is estimated between 4 and 10% (6), a rate almost 50 times higher than that of the general population (5). In addition, BPD remains notoriously difficult to treat. No specific medication has been officially approved for its treatment (8), and appropriate psychotherapeutic treatments are not or hardly available (2).

This general picture is accompanied by a lack of explanatory models of BPD based on experimental results (9). The aim of this review is to lay the foundations of a new model inspired by recent developments at the intersection of social cognition and behavioral ecology. Specifically, we argue that the sense of agency—the experience of controlling one's own action and through them, the course of events in the outside world (10)—is an important dimension to consider when characterizing self-disturbances in BPD. Second, we address the well-known, though poorly specified, relation between self-disturbances and adverse conditions encountered early in life. We then highlight the potential relevance of the phenotypic plasticity framework in evolutionary biology [i.e., Life History Theory (11)] to make sense of this association. We hypothesize that the effect of early life adversity on BPD symptomatology is partly conditional on the way individuals trade their limited resources between competing biological functions along the life cycle.

### The Sense of Self in BPD

The DSM 5 mentions disturbance in the sense of Self as being central to BPD (12). Kernberg, a prominent clinical theorist of BPD, proposed the concept of “identity diffusion” (13) to refer to difficulties in establishing and maintaining a stable and coherent sense of Self. Identity diffusion is signed by the expression of fragmented states of mind about the self, such as “*having great difficulty in answering simple (but psychologically essential) questions like: Who am I? How can I be differentiated from everybody else? What do I want? How can my present life and problems be meaningfully related to my past history and my conceptions of the future?*” (14). Other formulations include the “painful sense of personal incoherence” (15). International classifications from DSM 3 to DSM 5 also refer to a broad phenomenological sense of discontinuity in BPD.

Consistent with these descriptions, results from qualitative studies (16–18) as well as experimental studies of patients' subjective experience [see for examples (19–24)] have shown that BPD patients have (i) impaired self-image, (ii) difficulty remembering childhood memories, (iii) lack of insight (25), (iv) difficulty projecting themselves into the future, resulting in the subjective experience of “living from day-to-day” (18, 26). Experimental results also frequently point to negative subjective self-evaluation in adolescents and adults patients (e.g., ideas of inner worthlessness and/or badness) [see (27) for a review].

#### Limitations of the Main Conceptions of Self-Disturbance in BPD

Despite many theoretical developments, past approaches to self-disturbance in BPD patients have encountered several limitations. Firstly, most studies referring to self-disturbance in BPD describe it as a disruption of the “sense of Self,” and use a wide range of concepts (28). There is little agreement on what the term “sense of Self” actually refers to (14). This gap is reinforced by the lack of consensus on the true meaning of Self in cognitive neuroscience and philosophy. Indeed, the concept of Self is multifaceted, yet it is often used as an umbrella term for notions that only very partially overlap—e.g., *cognitive self, core self, dialogic self, ecological self, etc*. (29). Secondly, most studies on BDP have explored high-level cognitive processes related to self (e.g., linguistic representations of the self) (30).

An alternative notion of the self has been developed as an immediate sense of ownership of our experiences, that is, a sense of self that exists prior to any reflection. Contemporary philosophers refer to it as a kind of minimal self (31), rooted in bodily sensorimotor processes. There is now agreement within the literature on a general dichotomy between (i) this minimal/embodied self, understood as a first-person pre-reflective experience implemented in bodily sensorimotor processes, and (ii) a higher-level self, grounded in reflective processes involving beliefs, introspection, and autobiographical memory (32).

Proponents of the embodied cognition theory have long argued that these two types of “self” are related. In this view, the body plays an essential role not only in perception but also in higher cognitive abilities (e.g., language, sociality, abstraction, intentional action, volition, creativity, and innovation) (33). In addition, embodied self has also recently been emphasized in computational models of the self-relying on a predictive coding framework (34). In this framework, representations of the self as well as self/world boundaries are generated through top-down and bottom-up predictions modulating the multimodal integration of sensory inputs. Several authors further refer to this type of hierarchical self-model to explain disrupted self-representation in experimental settings as well as in a range of disorders including schizophrenia and depersonalization disorder (30, 35).

Some authors suggest that this embodied minimal self, combined with models developed in the predictive coding framework, hold promise to develop testable explanations of self-other disturbances in BPD (36). We review and critically discuss these recent theoretical developments in the next section.

#### The Minimal Self in BPD

BPD patients suffer from disrupted body experiences. These include “dissociative symptoms” (37). Although dissociation is a broad concept, including “flashbacks,” “identity confusion,” and a feeling of “having large gaps” in one's memory, it is commonly referred to as the phenomenon of depersonalization and is characterized by out-of-body feelings. These symptoms are part of the ninth diagnostic criteria of BPD in DSM V (“*transient, stress-related paranoid ideation or severe dissociative symptoms*”). In addition, a majority of BPD patients have negative self-assessments of their own body (38), higher pain thresholds (39), and non-suicidal self-injurious behaviors (2, 40).

To date, few studies have focused on the minimal self in BPD. According to the consensual multimodal account of the minimal self, we experience our body through our internal and external senses. These senses can influence each other, meaning that “*information from one modality can affect information in another modality*” (41). This has been demonstrated by several experiments in healthy subjects, showing for example that specific auditory information alters the feeling of the texture of our own skin [see also (41) for a review of the ways in which vision affects proprioception and touch].

The Rubber Hand Illusion (RHI) task (42) is a classic experiment investigating the multimodal binding account of minimal self. The experimental set-up is as follows: “*One sits with one's arm hidden behind a screen, while fixating on a rubber hand presented in one's bodily alignment; the rubber hand is then stroked in either synchrony or asynchrony with one's (hidden) hand (with cotton swabs for example)*” (41). This manipulation induces an illusion: (i) at the phenomenological level, healthy participants report that they experience tactile sensations as being located on the rubber hand, and that they feel as if the rubber hand belonged to them (i.e., illusory limb ownership); (ii) at the behavioral level, they mislocalize the stimulated finger in the direction of the location of the finger of the rubber hand (i.e., proprioceptive drift) (41).

Only three studies have investigated RHI in BPD patients. Two studies report an increased illusory limb ownership in adult women with BPD compared to healthy controls (43, 44). A third study using a modified version of the RHI did not replicate this finding (45). Interestingly, one of these studies showed that this increased illusion occurred not only in the synchronous condition, but also in the asynchronous condition (44). With respect to proprioceptive drift, both studies report similar drift toward the rubber hand in the synchronous condition in BPD and healthy controls groups. Nonetheless, BPD participants also showed a sustained proprioceptive drift during the asynchronous condition in one study (44). In addition, state and trait dissociations were both associated with illusory limb ownership (43). Since the sense of body ownership is based on tactile, proprioceptive and visual body percepts, the authors argue that the sustained illusory limb ownership and proprioceptive drift in BPD are due to an “*overweighting of exteroceptive input (e.g., seeing the rubber hand)*” to the detriment of interoceptive (i.e., tactile and proprioceptive) cues (44). Abnormal illusory experience through RHI have also been shown in schizophrenia, Body Dysmorphic Disorder and eating disorders as well as during ketamine challenge (44). As a result, RHI (43) as well as broader body ownership experiences, have been associated with state and trait dissociation (38) and trait psychoticism (44) in BPD subjects.

In addition to the notion of bodily illusion, the minimal/embodied self in BPD was also approached through the prism of interoception (46) or pain perception (39). Despite its potential importance in deficient self-awareness and psychosocial functioning in BPD (46), only two studies have investigated the involvement of interoception in BPD: one study found that interoceptive performance was not altered in BPD (47), while another provided neurophysiological evidence of a deficit in cortical representation of interoceptive afferences (i.e., heartbeat-evoked potentials) in BPD (48) [see (46) for a review]. With respect to pain perception, there is ample evidence that it is reduced in BPD (39). However, BPD patients do not show alterations in tactile and proprioceptive perception compared to healthy controls [see (46) for a review].

#### Does Minimal/Embodied-Self Explain Self-Disturbances in BPD?

The above studies provide converging preliminary evidence that BPD is associated with perceptual abnormality (i.e., pain perception), altered bodily awareness and disturbance of self-location (i.e., interoception, illusory limb ownership). Thus, it is tempting to speculate that BPD is related to the disruption of low-level perceptual and bodily processes. Indeed, several authors have repeatedly postulated that the general symptomatology of BPD is influenced by abnormal bodily processes that blur the boundaries between the inner (the self) and the outer (the environment, other people, etc.) (38, 43, 46). Can these alterations of the minimal self in BPD patients explain impairments in higher-order cognitive functions? This hypothesis currently faces several limitations.

Firstly, empirical evidence of a direct contribution of the disturbed minimal-self to BPD symptomatology, including disturbed psychosocial functioning, is scarce. Moreover, the few studies that address this issue lack statistical power or are gender-biased (i.e., often only females are included). Women are more likely to report suicidal or self-mutilation behaviors, affective instability, and chronic feelings of emptiness at lower levels of BPD severity than men (49). In contrast to the studies cited above (43, 44), another study failed to show an association between body experiences and childhood trauma, as well as major symptoms such as engagement in non-suicidal self-injurious (NSSI) behavior, and emotion dysregulation (38). Overall, the most robust finding is the relationship between alteration of the minimal self in BPD and dissociation (38, 43, 44).

Second, alterations of the minimal self similar to those reported by BPD subjects, have been associated with other psychiatric disorders (46). Similarly, increased illusory limb ownership has also been associated with other psychotic conditions (44). These findings call into question the specificity of these alterations.

**Figure 1:**
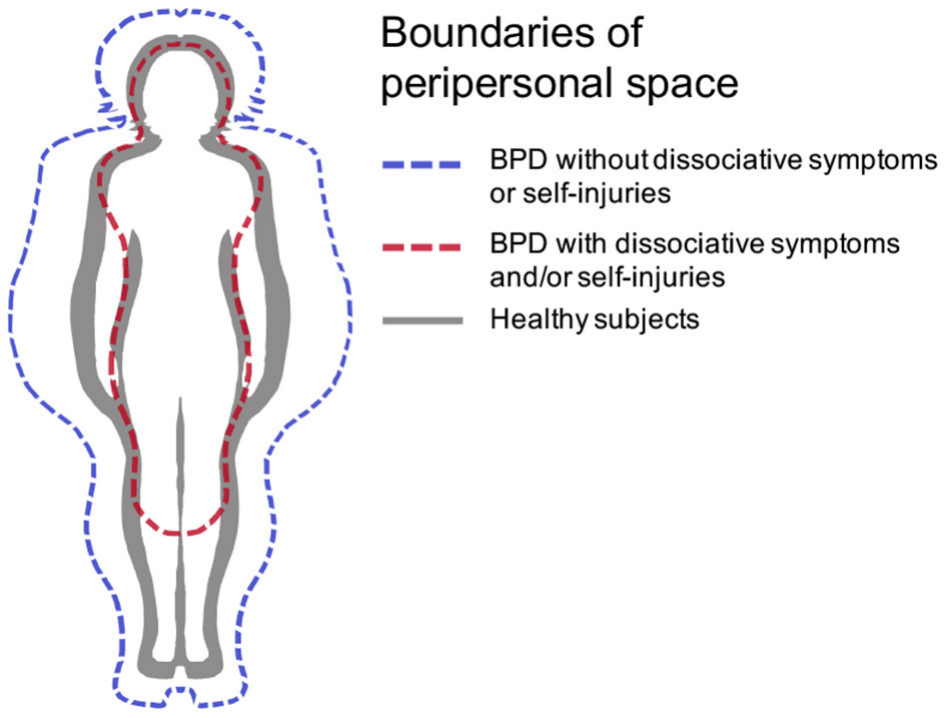
Hypothetical boundaries of peripersonal space in BPD [adapted from (41)], in the case where it is disrupted (red dashed line) or enlarged (blue dashed line).

Third, previous studies of the sense of body ownership in BPD may have missed an important affective dimension. De Vignemont proposed that the sense of bodily ownership cannot be reduced to body feelings and bodily attention (41). Indeed, bodily ownership is linked to the mental representation of body boundaries, which correspond to a safe zone that “*requires appropriate actions if it is invaded*.” This body map is a response to organism's evolutionary needs and defines a specific representation of the “narcissistic” body that must be protected (protective body schema). This theoretical development shed new light on the experimental results on illusory bodily ownership in BPD: the more unstable body representation in the mind revealed by increased RHI in BPD could be interpreted as a precarious protective body schema. Interestingly, in the stop distance paradigm, adult women with BPD have twice the preferred social distance compared to healthy subjects, confirming previous experiments that established a preferred social distance in imagined social experiences (50). In the light of de Vignemont's theory, these results suggest an enlargement of the protective body schema in BPD (see Figure 1). Note, however, that this interpretation seems to be contradicted by the high prevalence of self-injuries in this disorder, which is most likely related to a disrupted protective body map (51, 52). This contradiction may be explained by the lower stability over time of the protective body map in BPD, which is itself linked to the particularly unstable affective state in these patients (53, 54).

#### Preliminary Conclusion

To overcome the above limitations, future research in BPD should focus on better characterizing the relationship between bodily experiences and interpersonal difficulties, and on the role of affective experiences (particularly those related to the protective body schema). In order to objectify these associations, future studies could combine experiments with neurophysiological measures, such as skin conductance, to capture physiological response to a potential threat.

BPD appears to be best conceptualized as a multifaceted disorder of self-experience, related to the disruption of narrative and minimal/embodied aspects of the self. The challenge for future research would be to unravel the interplay of emotional instability, bodily experiences and impaired interpersonal functioning in BPD. Specifically, there is a lack of accurate understanding of the disrupted social relationships in BPD, which are considered a central dimension of the disorder.

### Self and Interpersonal Functioning Impairments in BPD

Consistent with the inclusion of both “perception and understanding of self” and “perception and understanding of others” in the “social processes” domain of the National Institute for Mental Health RDoC (55), most theories of BPD have linked self-disturbances in BPD to interpersonal problems (1–3, 20). Kernberg proposed that self-disturbance is related to a pattern described as intrapersonal and interpersonal “splitting” that involves polarized or extreme evaluations of self and others (13). The notion of “dichotomous thinking” also describes the tendency of BPD subjects to evaluate their experiences in terms of exclusive categories (56). Empirical studies have supported the view that BPD involves split evaluations of others (20, 57, 58) and that individuals with BPD perceive close relationships in “*extremes of idealization and devaluation*” and alternating between “*overinvolvement and withdrawal*” (DSM-V, 2014). Since then, previous research has provided evidence of impaired ability to recognize and reflect on mental states of self and others in BPD (59–61). Similarly, Bateman and Fonagy proposed the concept of “alien self” in BDP individuals to describe how they experience being controlled by external forces (62), while Gunderson proposed the theory of interpersonal hypersensitivity in which “*the BPD person reacts to perceived failures of support from others by feeling either that this is cruelly unfair (“bad other”) or that he or she is inherently bad (“bad self”)*” (63). Indeed, mistrust is an important characteristic of interpersonal problems in BPD. Studies using explicit as well as implicit measures such as those used in game theory paradigms (64), evaluative priming tasks (57) or live dyadic interactions (50), have shown that BPD patients tend to consider ambiguous or neutral faces as more angry, and interpersonal stimuli as more negative in general (65, 66). These tendencies may explain why suicidal behaviors are so common among BDP individuals (67).

In summary, BPD patients appear to struggle with negatively biased and conflicting mental representations of self and others. According to Gregory (68), this is reminiscent of an unstable binary attribution of “values,” “agency,” and “motivations” (68). In other words, in BPD patients, the locus of control— i.e., the degree to which individuals believe that they, as opposed to external factors such as chance, fate, or other agents, have control over their lives (69, 70)—appears to be shifting from a position of no responsibility to full responsibility for an interpersonal event. To our knowledge, only a few empirical studies have examined the attributive style of BPD. Two psychometric studies suggest that BPD characteristics are associated with an external locus of control (71, 72). Other studies have shown that patients with BPD appear to consider themselves to be the predominant cause of adverse events (73, 74). These results, combined with recent attention to BPD patients' mistrust of others (75), support the hypothesis that BPD is associated with poor attribution of actions and intentions to agents. These misattributions participate in the blurring of boundaries between self and others, and may ultimately result in an abnormal experience of agency in BPD patients.

## The Acting Self: Why Agency is Important

It has long been suggested that self-other discrimination requires a functional sense of agency (SoA), that is the unaltered experience that “I am the one who is causing or generating the action” (76), and through them, the course of events in the outside world (10, 77). In our daily lives, we most often experience agency as an experience that is compatible “*with a description in terms of degrees of control over our actions*” (76) and their subsequent outcomes. It is important to note that the consequences of the intended action may be proximal (e.g., turning on the light) or distal (e.g., having provoked a particular emotional expression in another person). Research on SoA has shown that the agentive experience is not a unitary construct and relies on a variety of information and neurocognitive processes.

### Implicit vs. Explicit Sense of Agency

**Figure 2:**
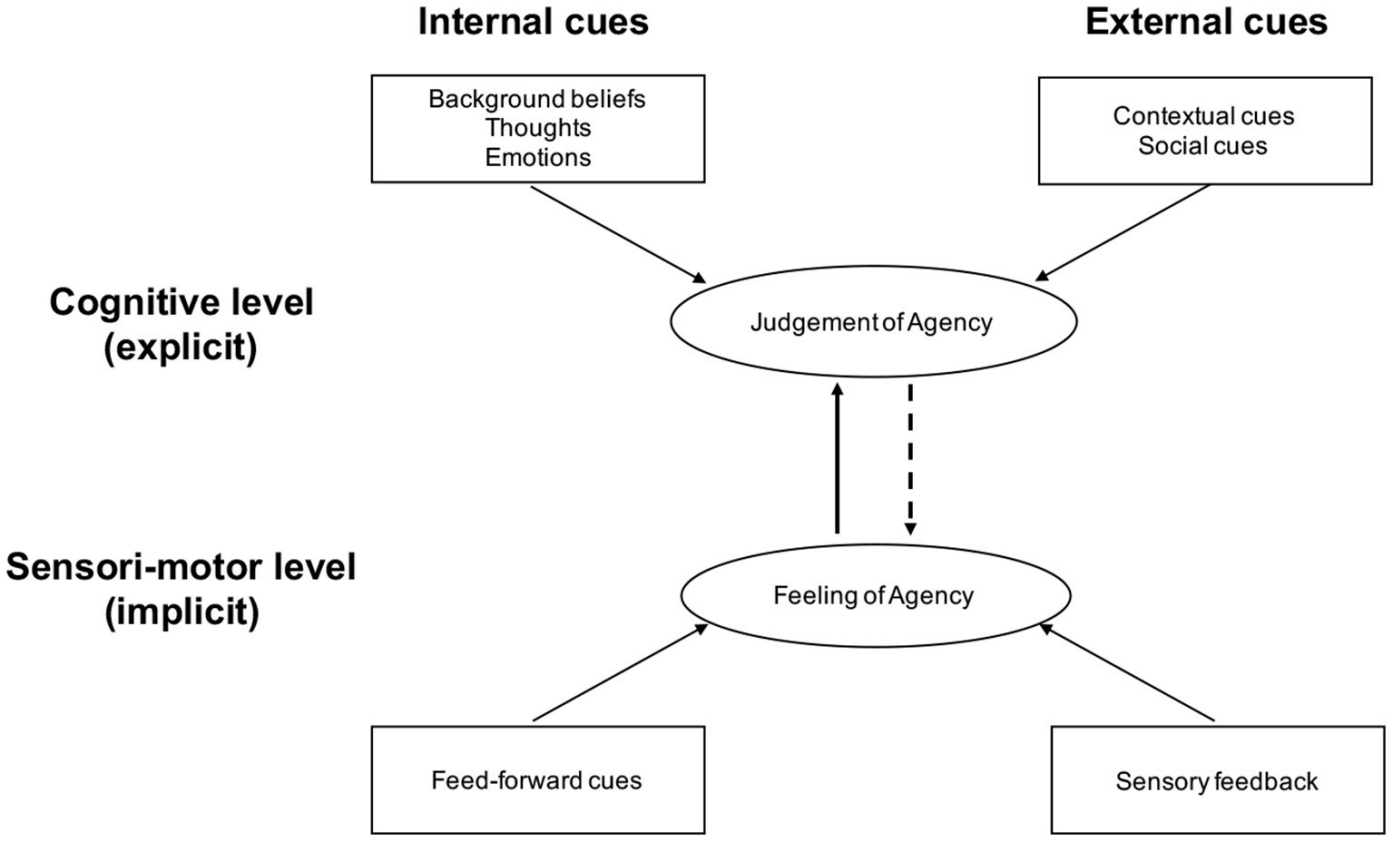
A cue integration model of SoA [adapted from (79, 80)].

The distinction between reflective and pre-reflective self-awareness that we mentioned earlier about the self also applies to the Sense of Agency (SoA). The current cognitive model distinguishes between (i) an implicit SoA, mediated by lower-level pre-reflective sensorimotor processes underlying the processing of action (78), which are highly sensitive to the discrepancy between intended and observed action effects, and (ii) an explicit, declarative or conscious SoA, which is based not only on pre-reflective processes but also on abstract self-knowledge and autobiographical memory, generally referred to as belief-like processes (79). Modern theoretical frameworks have proposed a taxonomy of the different contributors to SoA, and emphasize that the SoA results from the integration of information from multiple sources (76, 80) (see Figure 2). At the lower sensori-motor level, in healthy subjects, recent studies have shown that the SoA depends on actively monitoring various internal signals related to the different stages of action control (i.e., prediction, actions selection, efference copy) (81), as well as external cues such as sensory inputs from the environment (35). At a higher cognitive level, SoA depends on monitoring internal signals such as explicit prior intentions, higher order beliefs, as well as external signals that relate to the general context of the action, such as social information (82, 83). Dimaggio and colleagues also coined the concept of existential agency (84) and proposed a distinction between what they called “instrumental agency” (that is “*agency in dealing with the non-personal world*”) and “interpersonal agency” (“*which involves affecting how others think and behave*”) (85).

Nevertheless, the broad dichotomy between implicit and explicit SoA should not be seen as an absolute dichotomy, but rather as a continuum. Thus, implicit or non-conscious mental representations can influence explicit SoA, as illustrated by a study showing that an unconsciously preactivated emotion increases the feeling of having caused the same emotion in another agent (86). Conversely, an explicit mental representation can influence implicit SoA, as illustrated by another study showing that recollection of an episode of social exclusion can alter the estimated interval between action and effect, the latter being a standard measure of implicit SoA (87).

SoA has been shown to be of particular importance for different facets of the self. In particular, it has been shown to modulate the sense of body-ownership (88). It may also be essential for the constitution of a narrative self, as the self as a “doer” would be necessary for the construction of the diachronic self as a “story teller.” In the words of Elisabeth Pacherie: “*a sense of oneself as an agent apart from any particular action, i.e., a sense of one's capacity for action over time, and a form of self-narrative where one's past actions and projected future actions are given a general coherence and unified through a set of overarching goals, motivations, projects and general lines of conduct*” (89). Finally, SoA plays an important role in social cognition, and in the regulation of interpersonal transactions, insofar as it indexes our ability to distinguish self-generated events from externally generated events (90).

Since SoA depends on a wide range of factors and develops throughout lifetime, it is not surprising that not all people experience it in the same way. Some people have great difficulty knowing what is self-produced and what is externally produced. Specifically, SoA can be disrupted in multiple ways. It can be disrupted by problems with high-order introspective cognition and/or by a failure to integrate sensory and motor signals (76). Using a transnosographic perspective, Dimaggio et al. proposed that impairments in self-reflection might be an interesting dimension to better capture and delineate SoA impairments in psychopathology. The authors posit that these impairments could be related to an altered sense of ownership of one's own thoughts and actions, poor emotional awareness, confusion between fantasy and reality, and poor integration of a range of self-representations and autobiographical narratives (84). Besides, experimental evidence suggests that SoA disorders in patients with schizophrenia are caused by a primary deficit at the sensorimotor level (91).

With respect to BPD, minimal and narrative self-disturbances as well as social impairments (especially attribution problems) could be associated with an altered SoA. A few authors have pointed in this direction [see for example (14, 19, 67, 92)]. More recently, Gold and Kyratsous have speculated that self-disturbances in BPD have both diachronic (i.e., narrative) and synchronic (i.e., agentive) components (93). Also, studies have shown poorer SoA in individuals with vulnerable narcissism (94, 95). Two recent experiments in BPD show contrasting results: an experiment using the Sensory Attenuation paradigm suggest that this implicit measure of SoA is abnormally decreased in a subgroup of BPD patients without non-suicidal self-injury (96). Another experiment using an intentional binding paradigm incorporated into a modified RHI paradigm provided evidence for higher self-reported SoA, but no conclusive evidence of an impaired implicit measure of SoA (45). However, these preliminary results should be interpreted with caution given their methodological limitations and lack of statistical power.

### Internal vs. External Sources of Information

The optimal cue integration model of SoA could help shed new light on SoA disruptions in BPD. As mentioned above, this model emphasizes the contribution of various sources of information to the experience of agency (80). One way to categorize these information sources is to distinguish between: (i) internal information that encompasses various signals related to the different stages of action control (81), and (ii) external information that relates to the context of the action, e.g., social information (82, 83) (see Figure 2).

In the “cue integration” approach, the respective contribution of internal vs. external information to SoA depends on their current reliability. In this approach, an “optimal” SoA is therefore the product of an “optimal” weighting of each source of information at a given time. For subjects with BPD, this adaptive weighting may precisely deviate from optimality. An interesting, though unexplored, possibility is that of an unbalanced contribution of internal vs. external information among BPD subjects: an underweighting of internal cues could mechanically increase the effect of external cues on SoA.

The idea that the SoA of BPD individuals may be particularly prone to modulation by external information is further fueled by their hypersensitivity to social information (2). It follows that sense of agency (SoA) in BPD patients could depend excessively on social context [for studies showing that social interactions modulate SoA in healthy adults, see (82, 83)]. Patients with BPD may struggle with the feeling that their actions (including their mental actions) depend on the physical and mental actions of others. In other terms, the boundary between self and others could be blurred and result in BPD being constantly overwhelmed by others, being represented alternately as potential satisfiers of an exacerbated need for care or as potential perpetrators (97).

Such a blurring of self-other distinction could trigger occasional misattributions of agency in BPD patients. It may explain altered bodily experiences, such as depersonalization, as SoA have been shown to be a key contributor to the sense of body ownership. Moreover, it could also have major implications for emotional regulation, a crucial dimension of the disorder (2). BPD patients suffer from an increased physiological reactivity to emotional situations, as well as a low emotional awareness (an inability to distinguish emotions) and an inability to implement effective emotion regulation strategies in order to reduce negative affects [see (53, 54) for reviews]. Nonetheless, the mechanisms underlying emotional distress in BPD are not fully known (54). Research has shown that causal attribution of arousing events have a strong effect on emotional experiences in the general population (98). Misattribution of arousing events may bias emotional experiences in BPD, further hindering the appraisal of these emotional experience and the implementation of effective emotional regulation strategies. For example, in the context of intense emotional experiences, which are typical of BPD, an illusory sense that an agent has caused their own positive affect could induce overly positive “other-agency emotions” such as excessive gratitude. Conversely, misattributing a negative affect to another agent could result in inadequate negative “other-agency emotions” such as anger. The sense of having caused a negative affect in another agent could also result in inadequate negative emotions such as guilt. Depending on the affective valence, positive or negative, of BPD patients' current relationships with others, misattribution of hyperarousing events may further explain why they alternate between extremes of idealization and devaluation of others and self (24), and between hostility and submissiveness (99). In stressful situations, misattribution may also explain these patients' paranoid ideations. In turn, the increased dependence of SoA on such volatile and contradictory representations of others may explain the difficulty for BPD patients to maintain a “stable and coherent sense of self.”

## Why Ecology Matters

Abnormal experience of agency in BPD patients may interact with the context, past and present, in which the disorder developed. This relationship between contexts—in the broadest sense: family, emotional, socioeconomic—and self-agency has been pointed repeatedly, but the question remains as to why this interaction occurs.

A large body of research suggests that representations of agency develop gradually over the first years of life, as we interact with the environment and learn to recognize ourselves as an agent among other agents and as a self among other selves (100–102). This complex feedback loop involving both the infant and their social environment is nonetheless fragile and thus vulnerable to virtually all forms of external perturbations. Its disruption could—possibly through a cascade of developmental events—precede the under- or over-estimation of the degree of control an agent can exert in a particular context, resulting in learned helplessness and delusions of control later in life (102).

Borderline Personality Disorder can indeed be conceptualized as a particular outcome of a developmental cascade that originates in an early experience of adversity—that is, of external events that threaten the individual's survival [(103); for a review, see (104)]. Along with genetic makeup [heritability is estimated to be ~40–60%, see (105, 106)], some early life environmental factors of adversity have indeed been robustly associated with BPD [for a review, see (104)]. For instance, low socio-economic status, poor or inconsistent parenting, physical violence, sexual abuse and emotional neglect have been precisely targeted as critical risks of expressing BPD at both the adolescence and adulthood [(107); for a review see (104)].

Even though this issue has not yet been directly studied, some recent findings suggest that early life adversity may also play a role in the emergence of agency-based symptoms. For example, there is some evidence to support the hypothesis that early life adversity promotes the belief of a diminished personal control over one's life (108).

People of lower social class, in possession of fewer resources, exposed to greater instabilities, or in a poorer health status, tend to perceive external, uncontrollable forces and other individuals as the primary causes of events that affect their lives [for a review, see (109)]. In contrast, people with higher socio-economic position perceived themselves (their internal states, motivations and emotions) as the primary causal sources of what happens to them (109). In addition, some recent works suggest that certain factors of early life adversity (e.g., economic scarcity, attachment insecurity) increases mental health problems (e.g., depression, anxiety) later in life *via* an *external* locus of control (110, 111).

Despite these research efforts, however, no existing work has systematically investigated whether the effect of adverse environments on reduced personal control (e.g., reduced self-efficacy) can extend more broadly to abnormal sense of agency (SoA) that, we believe, is a common correlate of BPD. To the best of our knowledge, only one recent study found an association between a factor of adversity—social exclusion—and a standard implicit measure of SoA called “intentional binding,” i.e., the compression of the perceived time interval between a voluntary action and its effect (87). This compression has been positively associated with the declarative sense of being the cause of the outcome of an action, and is considered a key signature of agency (112).

## Why More is Needed: Life-History Trade-Offs

**Figure 3:**
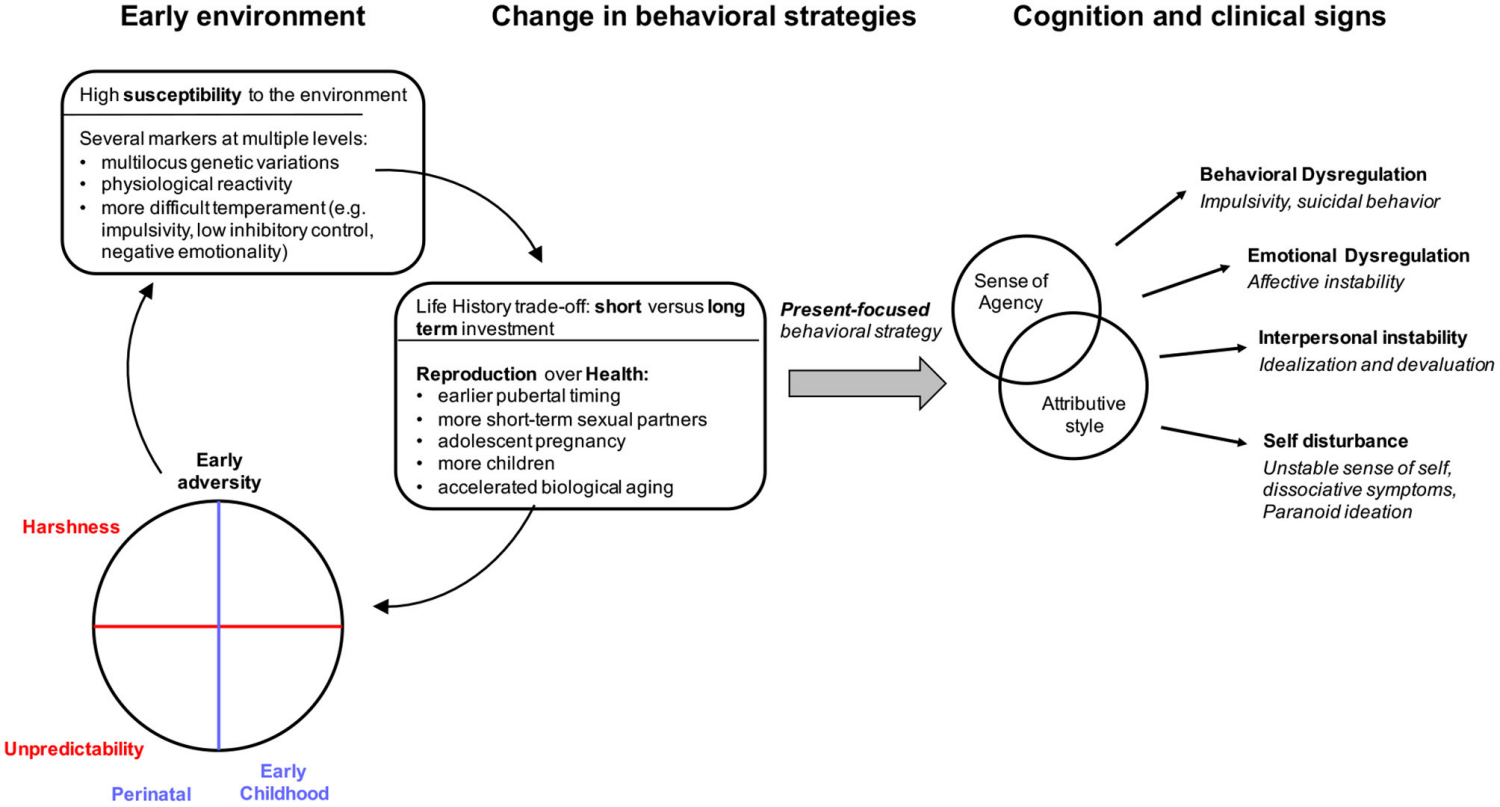
Putative developmental processes mediating the association between early environmental adversity and BPD condition.

Although some associations between early life adversity factors, the expression of BPD, and the emergence of agency-based symptoms have been independently reported in the literature, the proximate mechanisms that may underlie these seemingly distinct associations remain underspecified. An interesting possibility is that the expression of BDP symptoms might partly result from the negative effect that early life adversity imprints on individuals' perceived agency. In this final section, we suggest that this phenomenon is mediated by a trade-off between present and future goals that operates at both the physiological and the psychological level of an individual's biology (see Figure 3). This idea borrowed from Life-History Theory, a branch of evolutionary developmental biology that studies how organisms organize their life schedule to optimize their survival and reproduction by allocating their limited stock of energetic resources among competing biological functions (11). Individual differences in these allocation patterns are thought to depend on the differential susceptibility of organisms to modify their phenotypes in response to environmental stress through mechanisms of adaptive plasticity (113, 114) (see Figure 3).

Recent empirical evidence and formal models (115) posit that individuals who report experiencing little or no control over events that affect their lives perceive the future as uncertain. From a biological fitness perspective, it makes sense for these individuals not to risk delaying the satisfaction of immediate needs to invest time and energy in long-term activities whose pay-offs are likely to change unpredictably. Some empirical data appears to support this hypothesis by showing that people who have experienced environmental uncertainty or low perceived control are more likely than others to act on a day-to-day basis in various domains such as health, reproduction, social or economic decision-making (108, 116–125). Interestingly, such a present-future trade-off clearly appears to be at play in people with BPD (18). Several studies have indeed associated the BPD diagnosis with a domain-general sensitivity for short-term rewards: more substance abuse (126, 127), more sexual risk-taking (128–130), less social trust (131), less cooperation (64), strategies favoring short-term reduction in emotional stressors over long-term emotional stability (54), and low tolerance for delayed gratification (132).

Particularly characteristic of BPD people (133), this present-oriented strategy (as well as its putative psychological cause in terms of reduced sense of control) could represent the behavioral correlate of a trade-off operating at a deeper physiological level, in the form of a prioritized allocation of energetic resources to the development of reproductive functions at the expense of somatic maintenance functions.

Prioritizing reproduction can be conceptualized as a present-oriented life strategy because it carries direct and indirect health costs that could otherwise be minimized by delaying reproduction for females (134) or by mitigating reproductive efforts for males (135, 136). Although debated [see (137)], a growing body of human data supports this prediction. For example, early life adversity accelerates the timing of human reproduction while decreasing investment in health (138–140). In contrast, safe childhood conditions are associated with delayed reproduction (134, 141–144) and are negatively associated with a number of morbidity risks (145). In addition, a recent meta-analysis of 65 studies has shown that experiencing adverse events (particularly traumatic events) at an earlier age significantly accelerates the onset of puberty as well as biological aging (indexed by telomere length and age of DNA methylation) in both women and men (146). Finally, a recent study of large representative samples of the European population indicated that a decrease in perceived control over life events, as well as a psychological focus on the present time, were both related to a life-history trade-off characterized by poorer health status, younger age at first reproduction, and higher fertility (108).

A review of recent epidemiological data suggests that individuals with BPD may also display such a reproduction/maintenance trade-off (see Figure 3). For example, BPD individuals of both sexes report significantly more short-term sexual partners (130, 147). Women with BPD are also more likely to become pregnant during their youth, and have more children compared to other individuals (148). There is also evidence that earlier pubertal timing and accelerated biological age are potential moderators of the association between early life adversity and externalizing-internalizing behaviors (149). In line with these findings, a recent study among female adolescent BPD inpatients has shown that earlier perceived pubertal timing is associated with higher BPD symptoms (150). Finally, evidence suggests a major reduction in life expectancy in BPD due to illness, independently of suicide (2).

The reproduction/maintenance trade-off and its behavioral outcomes that appears to characterize both healthy and BPD populations might be partly determined in early ontogeny, when the developing organism must “decide” how to optimally allocate its limited energy budget between brain development and body growth (151). Importantly, the shape of these trade-offs depends, at least in part, on the environmental cues processed by the organism early in life. This is because building a biological system is a long and costly process, so a delayed allocation decision can be detrimental, and a reversal is often impossible. While economic deprivation, family instability, and physical insecurity can impact developmental trajectories primarily during early and late childhood (114, 115), low or unpredictable energy provisioning could have a disproportionate impact on developmental programming during the prenatal and postnatal periods where such a provisioning—and hence survival—is exclusively conditional on parental care (152). From this perspective, investing a large amount of metabolic resources in brain development is risky if food availability varies unpredictably, and slowing down neuromaturation to store more fat makes sense: it compensates for a potential lack of parental energy provisioning by relying on endogenous energy stores, even if it comes with costs to physical and mental health later in life, such as overweight, insulin resistance, reduced brain volume, cognitive impairment, or mental disorders such as ADHD (153–157). To our knowledge, no study has yet investigated whether a rapid increase in body growth between birth and childhood is a vulnerability factor for developing BPD symptoms later in life. Breastfeeding is thought to have a positive effect on maternal bonding and later attachment style (158). But more critically, it is possible that lack of breastfeeding, often reported in children who later developed BPD, is involved in adjusting the brain/body growth trade-off to inconsistent energy supply in the postnatal period (159).

Overall, much work needs to be done to show how BPD and agency-based symptoms are related to the life-history trade-offs described above (see Figure 3). Nevertheless, this new developmental approach offers some preliminary insights into the developmental pathway by which early experiences lead to alterations in SoA and, more broadly, to BPD symptoms.

## Conclusion

Overall, our perspective on BPD self-disturbances integrates and expands existing research and theories on BPD. It first suggests that the solipsist frame of reference of the “self,” whether narrative or minimal, cannot fully explain the core intersubjective dimension of BPD. Self and interpersonal functioning impairments in BPD are closely related to misattribution problems. These problems may involve attributing erroneous causes to one's own actions and emotions, but also to the actions and emotions of other agents. An approach that focuses on agency may allow researchers to track down the mechanisms underlying self and interpersonal disturbances in BPD. In addition, Life History Theory appears a promising framework to explain why, when and how agency-based symptoms develop, in the form of a biased locus of control or abnormal self-efficacy. This cross-disciplinary perspective provides the basis for a comprehensive explanatory model of the core symptomatology of the BPD condition.

Research at the interface of evolutionary developmental biology, social cognition, and psychopathology can accelerate the advancement of empirical knowledge about BPD, and these advances may have important implications for the prevention and management of the disorder. It could help patients to gain insight into the nature of their condition, and help clinicians to refine existing therapies for BPD. First, several authors have emphasized the importance of targeting altered SoA in personality disorders, including BPD (67, 160, 161). Second, the Life History trade-offs framework suggests that current psychosocial interventions for BPD might be more effective with a focus on global health. Advances in our knowledge of BPD developmental trajectory could enable the elaboration of specific intervention programs at each stage of development that would prevent the further development of BPD at a later age.

## Data Availability Statement

The original contributions presented in the study are included in the article, further inquiries can be directed to the corresponding author.

## Author Contributions

AB, PJ, and VC: conceptualization. AB and PJ: investigation and writing—original draft. AB, VC, and DC: resources, project administration, and funding acquisition. AB, PJ, VC, and DC: writing—review and editing. AB and VC: visualization. VC and DC: supervision. All authors contributed to the article and approved the submitted version.

## Funding

AB was supported by a fellowship from the Université de Paris. VC was supported by the Agence Nationale de la Recherche grants ANR-16-CE37-0012-01 (ANR JCJ) and ANR-19-CE37-0014-01 (ANR PRC). VC and PJ were supported by a department-wide grant from the Agence Nationale de la Recherche (ANR-17-EURE-0017) and by ANR-10-IDEX-0001-02 PSL (program Investissements d'Avenir).

## Conflict of Interest

The authors declare that the research was conducted in the absence of any commercial or financial relationships that could be construed as a potential conflict of interest.

## Publisher's Note

All claims expressed in this article are solely those of the authors and do not necessarily represent those of their affiliated organizations, or those of the publisher, the editors and the reviewers. Any product that may be evaluated in this article, or claim that may be made by its manufacturer, is not guaranteed or endorsed by the publisher.
